# A Curious Case of Colonic Perivascular Epithelioid Cell Tumor: A Unique Diagnosis With Variable Presentations

**DOI:** 10.7759/cureus.11164

**Published:** 2020-10-26

**Authors:** Joseph Bennett, Raquele Laury, Hongyan Dai, Charles Walde, Anup Kasi

**Affiliations:** 1 Department of Internal Medicine, University of Kansas Medical Center, Kansas City, USA; 2 Department of Pathology, University of Kansas Medical Center, Kansas City, USA; 3 Department of Oncology, University of Kansas Medical Center, Kansas City, USA

**Keywords:** pecoma, submucosal, colon

## Abstract

A 67-year-old female with a history of colon cancer underwent colonoscopy. An 8 mm semi-pedunculated, friable, and ulcerated lesion of the ascending colon was removed completely using a hot snare. Immunohistochemical staining showed strong positivity for transcription factor binding to IGHM enhancer 3 (TFE-3) and was partially positive for Human Melanoma Black (HMB-45), consistent with a diagnosis of perivascular epithelioid cell tumor (PEComa). The patient underwent endoscopic submucosal dissection of the residual lesion in the ascending colon without complications. Here, we discuss the clinical and histopathologic characterizations that helped guide the diagnosis and management of this exceedingly rare entity.

## Introduction

Perivascular epithelioid cell tumors (PEComas) are an incredibly rare and potentially malignant entity that can occur virtually anywhere in the body. First described in 1992, these tumors occur near vascular walls and express markers of melanocytic and smooth muscle differentiation [1]. Due to the rarity and limited studies regarding PEComas, this diagnosis can prove challenging for clinicians to risk-stratify patients and determine malignant potential.

## Case presentation

**Figure 1:**
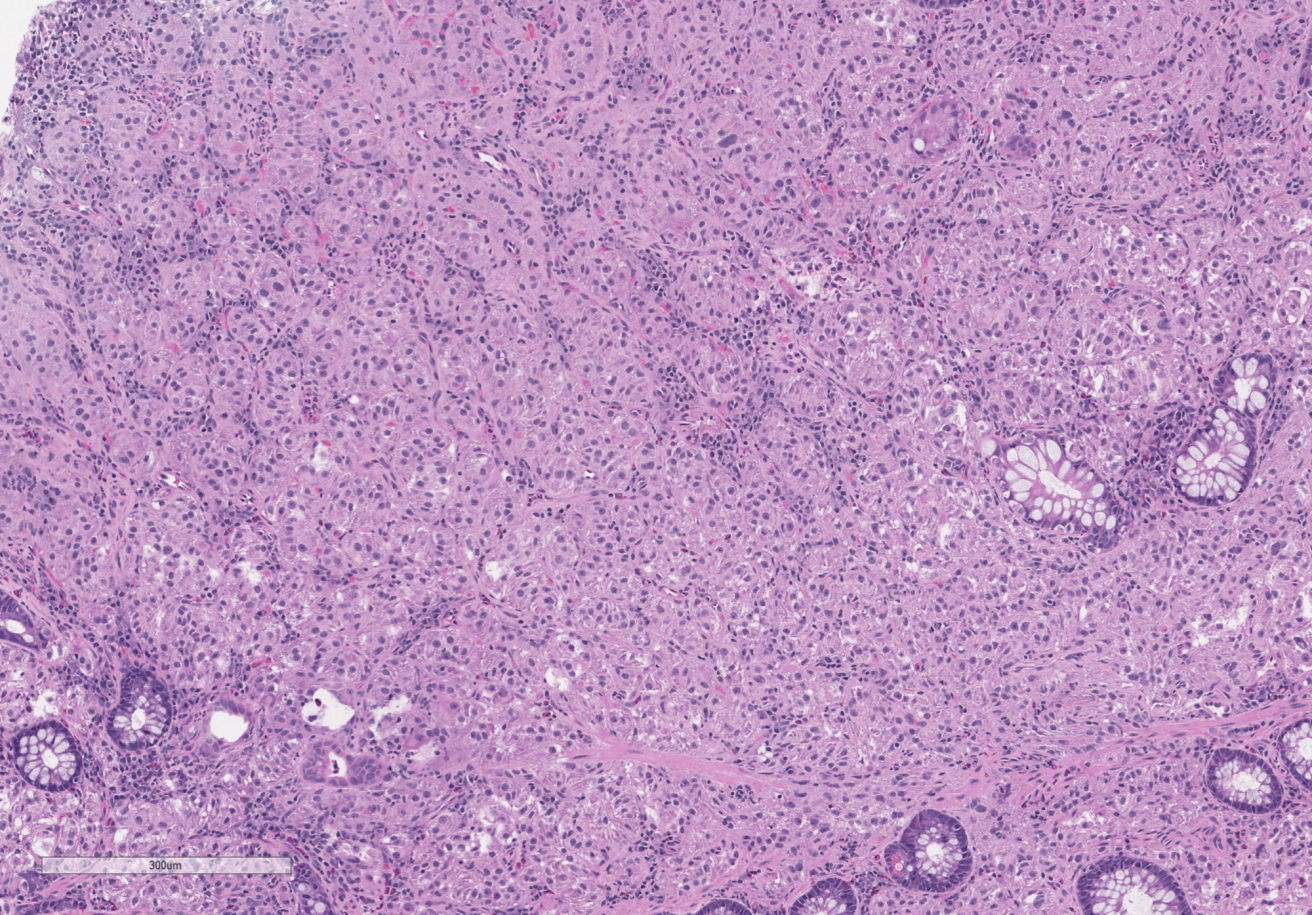
Routine H&E stained sections demonstrate epithelioid cells arranged in a nested pattern with granular eosinophilic cytoplasm H&E: Hematoxylin and eosin

**Figure 2:**
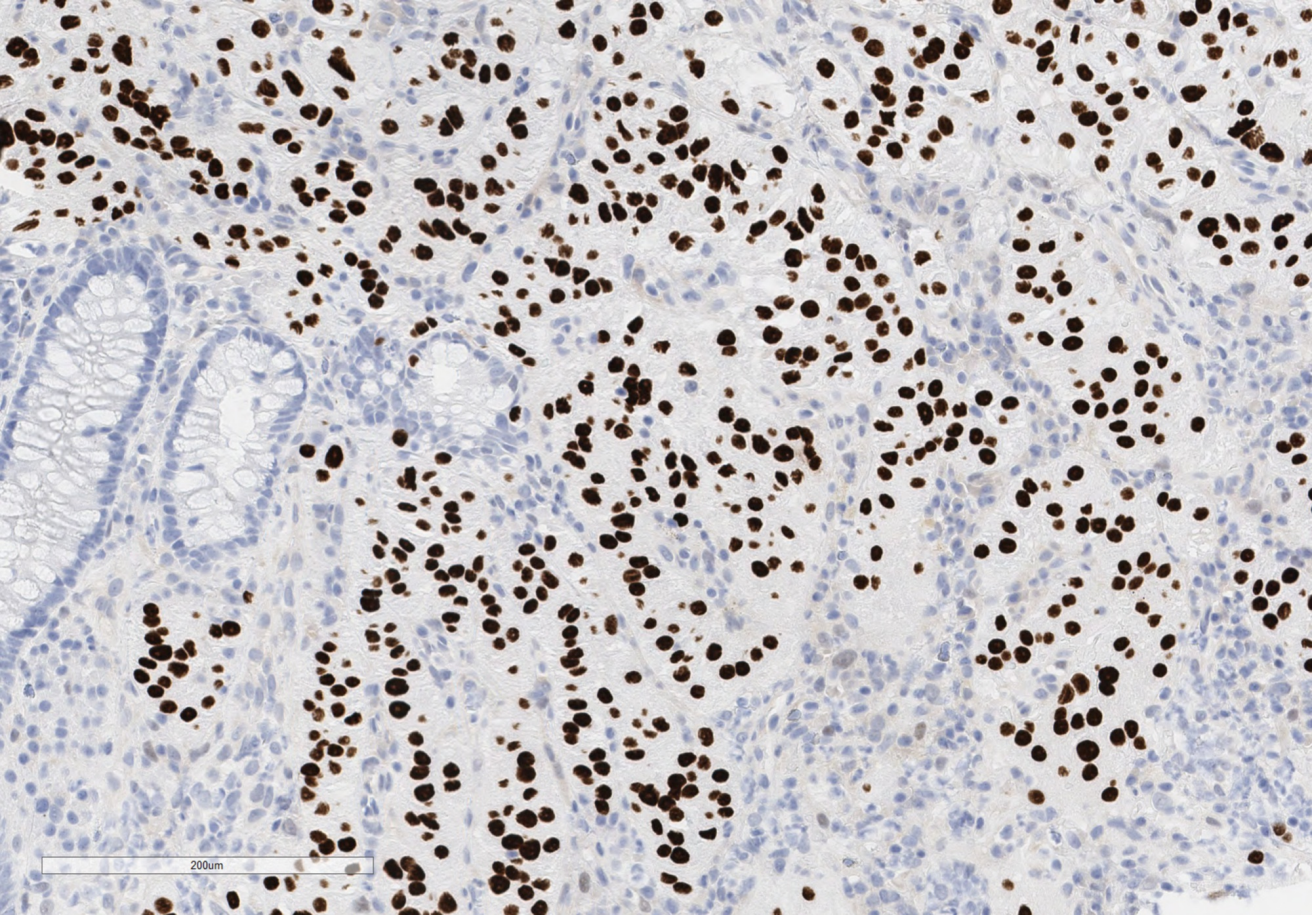
IGHM enhancer 3 (TFE-3) immunohistochemical stain demonstrates strong nuclear positivity

**Figure 3:**
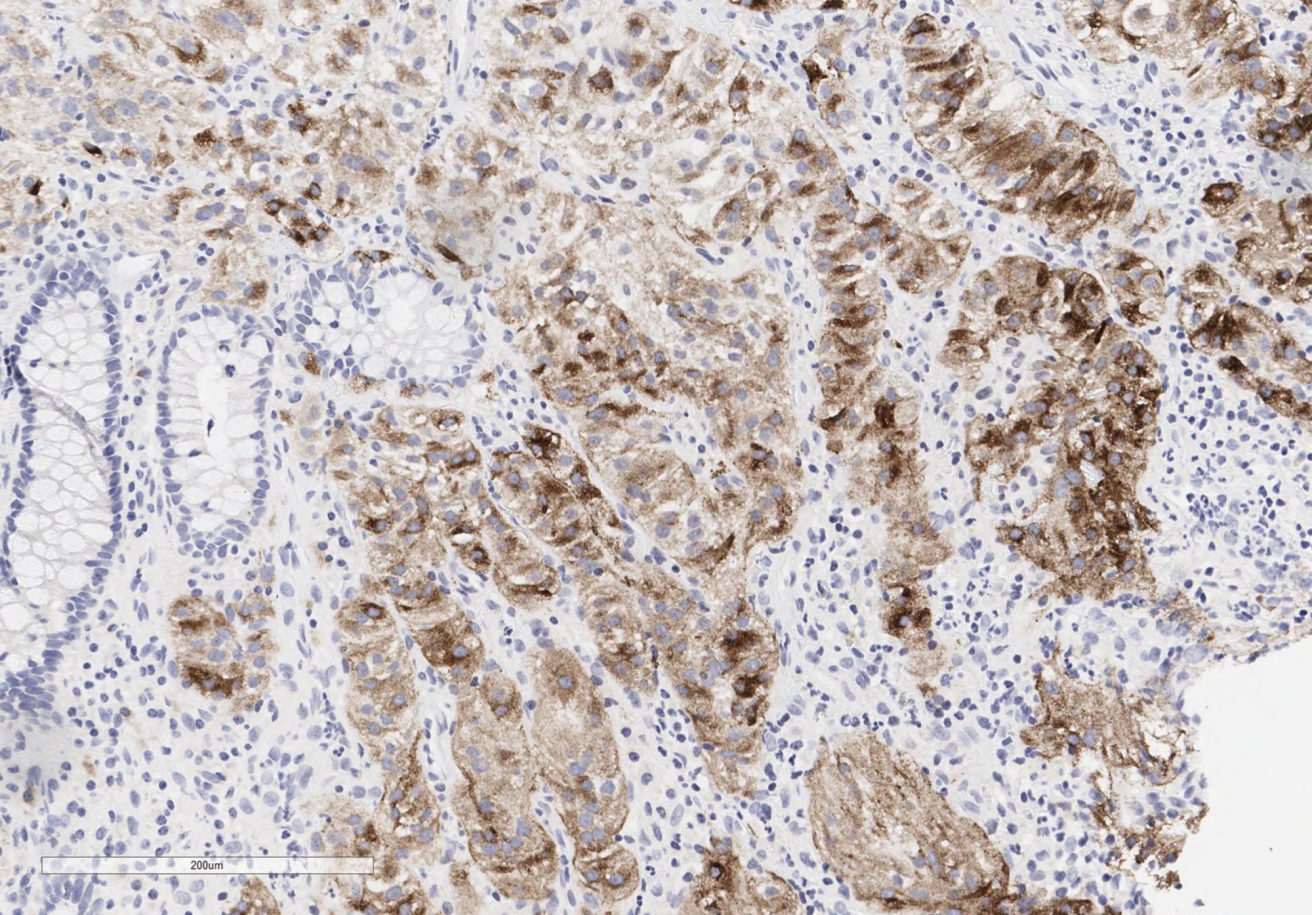
Human Melanoma Black (HMB-45) immunohistochemical stain shows partial cytoplasmic positivity

A 67-year-old female with a past medical history of colonic adenocarcinoma diagnosed at age 52 presented for screening colonoscopy. She had no gastrointestinal symptoms, weight loss, or clinical features of malignancy. Her physical exam was unremarkable. Complete blood count and general chemistries were within normal limits. On a surveillance colonoscopy, three polyps were found. An 8mm semi-pedunculated friable ulcerated lesion of the ascending colon was removed with a hot snare. A 5mm friable and ulcerated polyp in the ascending colon and a sessile 5mm polyp in the rectosigmoid colon were removed with a cold snare. While the histologic examination of the two polyps revealed tubular adenoma and hyperplastic polyp, the lesion removed by hot snare from ascending colon demonstrated nested epithelioid cell proliferation surrounded by a fine vascular network (Figure 1). Cells had abundant granular cytoplasm with occasional nuclear atypia and rare mitotic activity. Immunohistochemical stains showed strong nuclear staining for transcription factor binding to IGHM enhancer 3 (TFE-3 shown in Figure 2) and Human Melanoma Black (HMB-45) (Figure 3). The tumor cells were non-immunoreactive for smooth muscle actin, cluster of differentiation (CD) 31, Desmin, CD68, Mart-1, GATA3, chromogranin, synaptophysin, CD56, CDX2, SOX10, pancytokeratin, CAM5.2, S100, PAX8, and DOG1. Ki-67 staining showed a low proliferation index of less than 5%. These findings suggested a diagnosis of PEComa. Due to TFE-3 positivity, fluorescence in situ hybridization (FISH) studies were performed, demonstrating TFE3 rearrangement. However, TFE3 and ASPSCR1 fusion was not identified. CT imaging of the chest, abdomen, and pelvis did not show any evidence of nodal or distant metastasis.

The patient underwent repeat colonoscopy at the two-month interval for surveillance. At the site of prior polypectomy in the ascending colon, a 5mm nodule was noted. Complete removal was accomplished via endoscopic submucosal dissection (ESD), and resection and retrieval were complete. Repeat pathologic examination was negative for residual PEComa, which was confirmed by negative TFE3 and HMB-45 staining.

## Discussion

First described in 1992, PEComas consist of a rare and variable presentation that can occur virtually anywhere in the body [1]. PEComas comprise a family of tumors that includes angiomyolipoma, lymphangioleiomyomatosis, clear cell “sugar” tumor of the lung, and tumors displaying similar immunohistologic characteristics [1,2]. These tumors are classically associated with smooth muscle and melanocytic marker expression [3].

The histopathologic diagnosis of PEComa is often difficult as carcinomas, sarcomas, and melanomas are all in the differential. The correct diagnosis in this case was even more challenging due to positive TFE3 by immunohistochemistry. TFE gene alterations are most commonly associated with alveolar soft part sarcomas (ASPS) and Xp11 translocation-associated renal cell carcinomas (Xp11 TRCC), but rarely seen in PEComas. Morphologically, the tumor cells in PEComas are nested with clear and eosinophilic cytoplasm. This morphology bears resemblance to that of ASPS, which is characterized by pseudoalveolar arrangement of tumor cells with central dyscohesion and thick fibrous septae [4,5]. Both tumors can show rearrangement involving TFE3; however, ASPSCR1-TFE3 fusion gene is only seen in ASPS [5,6]. Xp11 TRCC is a distinct type of renal cell carcinoma which commonly demonstrates polygonal cytomorphology, a papillary and/or solid alveolar growth pattern with clear cells, psammoma bodies, and a high nuclear grade [7]. It is immunoreactive for paired-box gene 8 (PAX8) and can have the same translocation found in ASPS (ASPSCR1-TFE3). However, in contrast to a balanced translocation in Xp11 TRCC, the translocation in ASPS is unbalanced, leading to a loss of genetic material [8]. The absence of PAX-8 staining by immunohistochemistry, ASPSCR1-TFE3 fusion product by FISH, and renal mass on radiographic studies support a diagnosis of PEComa rather than secondary involvement by Xp11 TRCC. The negative staining for SRY-box transcription factor 10 (SOX10) and S100 rule out melanoma.

PEComas exhibit a wide range of clinical presentations from an indolent clinical course to mimicking aggressive soft tissue sarcomas [9]. To date, there is no consensus on the pathologic criteria to predict a more aggressive clinical course. However, pathologic features that may suggest malignant potential include marked atypia, increased mitotic activity, and necrosis [9,10].

In one study conducted by Doyle et al., 35 patients with gastrointestinal PEComas were studied for histologic characteristics and predictive factors of malignant behavior. Thirteen of 35 patients developed metastatic disease with a mean time to metastasis of six months . Their study concluded that the presence of metastasis was signiﬁcantly associated with the presence of marked nuclear atypia (P=0.0033), diﬀuse pleomorphism (P=0.02), and more than two mitoses per 10HPF (P=0.0002) [9]. In our case, these features were not present on histopathologic examination, hence definitive management by endoscopic submucosal dissection was pursued. Deeper lesions may require surgical resection. Regardless, risk estimation of metastasis remains difficult for clinicians due to the rarity of this entity. Thus, imaging was pursued to confirm the absence of secondary sites involved as well as repeat biopsy or resection to ensure complete removal of the lesion.

The primary treatment for PEComas and the prevention of local recurrence or distant metastasis includes resection of the lesion [10,11]. To date, this is the only potentially curative therapy. Preclinical studies have shown mammalian target of rapamycin (mTOR) inhibitors may help in controlling disease activity, including PEComas within the GI tract [12-14]. In one retrospective study, mTOR inhibitors were the most active agents in patients with advanced/metastatic PEComa compared to gemcitabine-based regimens, anthracycline-based regimens, and antiangiogenic regimens [15]. Unfortunately, PEComas are generally considered chemotherapy and immunotherapy insensitive [10,11,13].

## Conclusions

Due to the rarity of this diagnosis, consideration of PEComas in the differential diagnosis is not common. Submucosal lesions of the gastrointestinal tract may often be mistaken for gastrointestinal stromal cell tumors due to similarities in presentation. In sum, it is important to include PEComas in the differential of submucosal lesions to avoid misdiagnoses and delays in clinical care.
